# Global Co-regulatory Cross Talk Between m^6^A and m^5^C RNA Methylation Systems Coordinate Cellular Responses and Brain Disease Pathways

**DOI:** 10.1007/s12035-024-04555-0

**Published:** 2024-11-05

**Authors:** Oliver Chukwuma Orji, Joseph Stones, Seema Rajani, Robert Markus, Merve Demirbugen öz, Helen Miranda Knight

**Affiliations:** 1 Division of Cells, Organisms and Molecular Genetics, School of Life Sciences, University of Nottingham, Nottingham, NG7 2UH UK; 2 Department of Medical Laboratory Sciences, College of Medicine, University of Nigeria, Nsukka, Enugu State, Nigeria; 3 School of Life Sciences Imaging Facility, University of Nottingham, Nottingham, NG7 2UH UK; 4 Department of Pharmaceutical Toxicology, Faculty of Pharmacy, Ankara University, Ankara, Turkey

**Keywords:** Brain Disease, Cellular Response, Co-Regulation, N6-Methyladenosine, 5-Methylcytosine, RNA Modifications

## Abstract

N6 adenosine and C5 cytosine modification of mRNAs, tRNAs and rRNAs are regulated by the behaviour of distinct sets of writer, reader and eraser effector proteins which are conventionally considered to function independently. Here, we provide evidence of global cross-regulatory and functional interaction between the m^6^A and m^5^C RNA methylation systems. We first show that m^6^A and m^5^C effector protein transcripts are subject to reciprocal base modification supporting the existence of co-regulatory post-transcriptional feedback loops. Using global mass spectrometry proteomic data generated after biological perturbation to identify proteins which change in abundance with effector proteins, we found novel co-regulatory cellular response relationships between m^6^A and m^5^C proteins such as between the m^6^A eraser, ALKBH5, and the m^5^C writer, NSUN4. Gene ontology analysis of co-regulated proteins indicated that m^6^A and m^5^C RNA cross-system control varies across cellular processes, e.g. proteasome and mitochondrial mechanisms, and post-translational modification processes such as SUMOylation and phosphorylation. We also uncovered novel relationships between effector protein networks including contributing to intellectual disability pathways. Finally, we provided in vitro confirmation of colocalisation between m^6^A-RNAs and the m^5^C reader protein, ALYREF, after synaptic NMDA activation. These findings have important implications for understanding control of RNA metabolism, cellular proteomic responses, and brain disease mechanisms.

**Supplementary Information:**

The online version contains supplementary material available at 10.1007/s12035-024-04555-0.

## Introduction

Over a hundred and fifty different types of RNA modifications exist for rRNA, tRNA and mRNA species. With the recent high throughput characterisation of RNA methylation forms such as N6-methyladenosine modification (m^6^A), 5-methylcytosine (m^5^C), N^6^,2′-*O*-dimethyladenosine (m^6^Am) and other mRNA capping modifications, N1-methyladenosine (m^1^A), pseudouridine (Ψ) and dihydrouridine [1–8], there has been unprecedented discoveries in RNA biological regulatory systems. m^6^A modification is the most prevalent internal modification in mRNA and long non-coding RNAs (lncRNA) and has been shown to regulate mRNA nuclear splicing, translation efficiency and degradation [9–12]. It has also been implicated in numerous physiological processes such as stem cell differentiation, embryonic development, neuronal and glial cell function as well as synaptic plasticity [10, 13–17].

m^6^A modification is a reversible process moderated by m^6^A methyltransferases (writers), demethylases (erasers) and RNA-binding proteins (readers) which are commonly termed ‘effector’ proteins. In mammals, the m^6^A writer proteins, METTL3 and METTL14, form a complex in which METTL14 provides stability for the enzymatic reaction while the catalytic domain of the enzyme complex resides in METTL3 [18]. A third writer protein complex protein, the Wilms tumour-associated protein (WTAP), recruits METTL3 to the site of methylation and reinforces the binding of the enzyme to the substrate [19]. Other proteins involved in m^6^A methyltransferase activity include KIA1429, ribosomal binding motif 15 (RBM15) and its paralogue RBM15B, which act in concert with WTAP to provide stability and mRNA positioning for the methyl transfer and are, in addition, involved in nuclear RNA splicing and nuclear export [20–23]. An additional, newly discovered, m^6^A writer protein, METTL16, has recently been shown to be involved in the methylation of coding RNAs [24, 25] as well as non-coding U6 spliceosomal small nuclear RNAs [26]. Furthermore, ribosomal RNA can be m^6^A modified at two sites namely, *18S* rRNA at position A1832 and *28S* at position A4220a [27–29] which is catalysed by the writer protein METTL5 [30].

m^6^A readers comprise RNA-binding proteins which bind to m^6^A-RNAs localised in the nucleus where they play roles in RNA processing, mRNA decay, stability and export, or bind to m^6^A-RNAs in the cytoplasm where they are involved in mRNA transport, translation or degradation. They include the YTH domain-containing family proteins, YTHDF1, YTHDF2, YTHDF3, YTHDC1 and YTHDC2. The YTHDF1, YTHDF2, and YTHDF3 proteins have similar sequence identity and binding affinities toward preferred RNA motifs [31], and recent studies support dosage-dependent redundancy in their function to regulate m^6^A-dependent mRNA stability and translation [32–34]. The YTHDC1 protein is thought to be primarily involved in mRNA splicing and mRNA transport [35], while YTHDC2 has been associated with the efficiency of RNA processing and stability [36, 37]. The m^6^A demethylase eraser proteins, FTO and ALKBH5, belong to the AlkB subfamily of the Fe(II)/2-oxoglutarate (2OG) dioxygenase superfamily, and both require ferrous iron and α-ketoglutarate as cofactors. However, they differ in their mode of demethylation with FTO demonstrating oxidative demethylase activity in the long stem-loop domain of the C-terminus, while ALKBH5 directly removes methyl groups from single-stranded RNA non-oxidatively [38, 39]. Furthermore, in addition to m^6^A demethylation, FTO mediates tRNA m^1^A demethylation [40], whereas ALKBH5 is reported to be specific to m^6^A.

Methylation at the 5th cytosine carbon (m^5^C) is the second most commonly abundant RNA modification present in rRNA, tRNA and mRNA. Like m^6^A modification, m^5^C methylation influences post-transcriptional processes, translational processing, RNA stability and nuclear-cytoplasmic transport but in addition, owing to the diversity of RNAs m^5^C modified, can influence numerous other molecular functions [41, 42]. However, the ‘effector’ proteins, which influence m^5^C modifications and functional consequences, differ from m^6^A effector proteins. m^5^C methyltransferases include NOL1/NOP2/SUN (NSUN) domain family proteins, homologues of the DNA methyltransferase, DNMT2. NSUN1, NSUN2, NSUN5 and NSUN7 target rRNA, mRNA and tRNA [43–45]. NSUN1 and NSUN5 methylate cytoplasmic rRNAs, while NSUN2 and NSUN6 are involved in the methylation of mRNA type II cytoplasmic sites [46, 47]. NSUN6 and DNMT2 also target tRNAs for methylation at cytosine-72 (C72) and C38, respectively, while NSUN2 is also implicated in tRNA methylation at positions C34, C40, C48, C49 and C50[48]. In contrast, NSUN3 and NSUN4 are predominately located within mitochondria where they modify mitochondrial tRNA and rRNAs, respectively [49–51].

Removal of m^5^C modification is mediated by the ALKBH1, a pleitropic dioxygenase which equally demethylates m^1^A and m^3^C [52–54]. ALKBH1 has also been shown to mediate modifications of cytosine-34 (m^5^C34) in mitochondrial tRNAs (mt-tRNAmet) [49]. The TET1, TET2 and TET3 enzymes also demethylate m^5^C by oxygenation [55] and are involved in demethylation of DNA as well as RNA. Several RNA-binding proteins have been suggested to be m^5^C reader proteins. Nonetheless, the most well-studied m^5^C readers include the THO complex subunit 4 (ALYREF) which complexes with the methyltransferase NSUN2 and mediates mRNA transport in and out of the nucleus [56], and YBX1 which is involved in mRNA stabilisation [57] and mRNA splicing [58].

m^6^A and m^5^C RNA modification effector proteins are generally thought to be specific to each m^6^A and m^5^C RNA modification system. However, as m^6^A and m^5^C dependent post-transcriptional modification of RNA molecules continue to be characterised, their roles in the regulation of molecular and physiological processes within the same cellular subdomains have become evident. In addition, there have been recent reports that modification of one system facilitates or enhances methylation in the alternative methylation system along single transcripts. For example, Li et al. (2017) reported that within the 3’UTR region of a specific gene, cyclin-dependent kinase *CDKN1A* (p21), NSUN2 catalyses m^5^C modification, and METTL3/METTL14 catalyses m^6^A modification, and that both types of modification facilitated the methylation of the alternative modification form. Moreover, joint methylation at m^5^C and m^6^A synergistically enhances *CDKN1A* (p21) expression in cells that have been biologically perturbed, i.e. undergone induced oxidative stress [59]. A second study also recently reported that YTHDF2 can directly bind to synthetically modified m^5^C RNA probes although at a lower binding affinity than m^6^A modified RNA [60]. Furthermore, knockout of the *YTHDF2* gene revealed no changes to m^5^C abundance in mitochondrial RNAs (mtRNA), but rRNA m^5^C sites substantially increased globally. We hypothesised that m^6^A and m^5^C methylation may globally cross-regulate at the post-transcriptional level through modification of the other modification effector protein transcripts, i.e. transcripts that encode for effector proteins, as well as show functional interactions at the protein level by mutual co-regulation of pathways. To gain a better understanding of the potential for cross talk, we studied modification profiles of effector proteins, mass spectrometry protein co-regulation patterns of the RNA modifying effector proteins and enriched gene ontology pathways after biological perturbations. To confirm in vitro a functional relationship between a m^5^C effector protein and m^6^A-modified RNA, we assessed changes in colocalisation between m^5^C reader protein, ALYREF, and m^6^A modified-RNAs after activation of synapses in differentiated neuronal cells. Furthermore, we detail proteomic studies which indicate that ALYREF physically interacts with m^6^A protein machinery in the nucleus or in the cytoplasm.

## Materials and Methods

### m^6^A and m^5^C Modification Datasets and Protein Interaction Analysis

To examine whether m^6^A and m^5^C modification effector proteins are reciprocally regulated at the post-transcriptional level, we analysed m^6^A-sequencing data which mapped m^6^A sites at a 200–400 base pair resolution from human hippocampal adult white matter and grey matter, the brainstem (BS), cerebellum (CER), hypothalamus (HYP) and cerebrum (CEREB), as well as late-stage human foetal brain tissue [14, 61]. In addition, m^5^C-seq datasets generated by bisulphite conversion methods to identify m^5^C sites at a base resolution in human HeLa cells [56] and human brain frontal tissue [62] were interrogated. The locations of m^6^A modification across the entire transcriptome and approximate position within each mRNA were annotated to eight non-overlapping transcript segments using HOMER (Salk Institute, USA) and a combination of bioinformatics tools including bedtools v2.30 to convert file formats, as well as UCSC software tools [63, 64]. These segments were as follows: intron, exon, transcription termination site (TTS), transcription start site (TSS), 3’UTR, 5’UTR, non-coding, and intergenic. m^5^C writer, reader and eraser protein transcripts listed in Supplementary Table 1 were examined to determine if they were m^6^A-modified within particular regions of the transcript, e.g. 5’UTR, coding or 3’UTR, show multi-modification or differences in modification abundance across brain regions. Similarly, using the m^5^C sequencing data, m^6^A effector proteins were examined to assess the occurrence of m^5^C modification along each effector transcript and within specific regions of each transcript. We used the BIOgrid4.4. [65] database to identify proteins reported to physically interact with ALYREF. We included in our list of m^6^A effector proteins, proteins which have been reported to either be repelled, or attracted, to m^6^A binding sites [66]. We identified 15 studies [67–81] which employed co-fractionation, affinity capture-mass spectrometry (MS), affinity capture-western or proximity label-MS to characterise interacting proteins.

**Table 1:** Co-regulation of proteins associated with m^6^A and m^5^C RNA modification effector proteins as measured by isotope-mass labelling spectrophotometry changes in human protein abundance after biological permutation and enriched gene ontology processes associated with co-regulated protein pathways

		m^5^C co-regulation partners (percentile score)	Enriched terms for common co-regulated protein pathways
m^6^A effectors			
m^6^A writers			
	METTL14	NSUN4 (0.97), NSUN5 (0.92)	Mitochondrion
	METTL16	NSUN2 (0.89), NSUN4 (0.92), NSUN5 (0.91),	Protein phosphorylation, Mitochondrial processes, Ribonuclear protein processes, Transit peptide
	WTAP	ALYREF (0.99)	Ubiquitination, Viral transcription, SRP-dependent co-translational protein targeting, Cadherin binding
	RBM15	ALYREF (0.92)	SUMOylation, Intracellular ribonucleoprotein complex, Translation initiation
m^6^A readers			
	YTHDF1	NSUN2 (0.82), YBX1 (0.88)	Protein phosphorylation, Ubiquitination,
	YTHDF2	YBX1 (0.99), ALYREF (0.99)	Ubiquitination, Acetylation,
	YTHDF3	YBX1 (0.99), NSUN2 (0.90)	Protein phosphorylation
	YTHDC1	ALYREF (0.96)	mRNA splicing, Ubiquitination
m^6^A erasers			
	ALKBH5	NSUN4 (0.92)	Acetylation
		m^6^A co-regulation partners (percentile score)	Enriched terms for common co-regulated protein pathways
m^5^C effectors			
m^5^C writers			
	NSUN1	WTAP (0.80), YTHDF1 (0.73)	Protein phosphorylation
	NSUN2	METTL14 (0.79), WTAP (0.84), YTHDC1 (0.81), YTHDF2 (0.82), YTHDF3 (0.90), ALKBH5 (0.82)	mRNA splicing, Ubiquitination
	NSUN4	METTL14 (0.97), WTAP (0.84), YTHDC1 (0.83), YTHDF1 (0.82), ALKBH5 (0.92)	Ribosome, Membrane
m^5^C readers			
	ALYREF	WTAP (0.99), YTHDC1 (0.96) YTHDF2 (0.99), YTHDF3 (0.98),	rRNA processing, RNA splicing
	YBX1	YTHDF1 (0.88), YTHDF2 (0.99), YTHDF3 (0.99)	Ribonucleoprotein complex, Ubiquitination

The strength of co-regulation is denoted by the percentile score. m^6^A writers METTL14, METTL16, WTAP and RBM15 are co-regulated with m^5^C writers NSUN2, NSUN4, NSUN5 and the reader, ALYREF. The m^6^A readers YTHDF1-3 and YTHDC1 are co-regulated with m^5^C writer NSUN2, and readers ALYREF and YBX1. The m^6^A eraser, ALKBH5, is co-regulated with the m^5^C writer, NSUN4. m^5^C effector proteins NSUN1, NSUN2, and NSUN4 are co-regulated with the m^6^A effector writer proteins WTAP, METTL14; the readers YTHDF1-3, YTHDC1 and the eraser ALKBH5. The m^5^C reader proteins, ALYREF and YBX1 are co-regulated with the m^6^A writer WTAP, the readers YTHDF1-3 and YTHDC1

### Protein Co-regulation and Gene Ontology Analysis

To identify proteins co-regulated with both m^6^A and m^5^C modification effector proteins, and to investigate if there are interactions between the two sets of proteins, protein co-regulation analyses were performed using the ProteomeHD (https://www.proteomehd.net/proteomehd) software. ProteomeHD was developed to use isotope-labelling mass spectrophotometry to measure changes in human protein abundance following 294 biological perturbations [82]. Stable Isotope Labelling by Amino Acids in Cell culture (SILAC) experiments were used to generate data matrix report proteome fold-changes rather than absolute concentrations, mostly in whole-cell samples. The software employs computer algorithms to map functionally co-expressed proteins after biological perturbation based on a topological overlap measure and treeClust similarities. In this manner, co-regulation maps of proteins and associated functions can be characterised. Using a correlation cut-off ≥ 0.8 percentile score (PS), co-regulated proteins for each set of m^6^A and m^5^C effector proteins were assessed. Data for ALKBH1, DNMT2, NSUN3, RBM15B and METTL5 proteins were not available. We chose not to examine the m^6^A eraser FTO as this demethylase is also involved in m^1^A demethylation nor the TET demethylase proteins as they are involved in DNA methylation processes. The lists of the top 1000 co-regulated proteins per effector protein were subsequently examined to assess if effector proteins were indicated to be co-regulated with the alternative methylation system. We subsequently assessed if there was commonality in the functional processes associated with each effector protein co-regulation protein profile across the m^6^A and m^5^C effector protein systems. To study such functional characteristics, gene ontology (GO) functional analyses of the top 1000 co-regulated proteins per effector protein were performed using DAVID [83, 84]. However, if fewer than 10 proteins were enriched per GO term, these terms were discounted. Co-regulated proteins associated with a significantly enriched GO term identified as of interest between individual effector proteins and methylation systems were analysed using in-house R scripts to determine the number of proteins and percentage of overlap of proteins. Figures were created using GraphPad Prism 8 or with BioRender.com.

### Immunofluorescence Microscopy of Differentiated and Synapse-Activated Neuronal SH-SY5Y Cell Cultures

The human neuroblastoma cell line, SH-SY5Y (ATCC CRL-2266, Sigma-Aldrich 94,030,304), was cultured under standard conditions using HAM’s DMEM/F12 (1:1) Nutrient Mixture supplemented with 10% FBS and 1% penicillin/streptomycin (Sigma-Aldrich, UK). Cells were grown up to a confluency of 90% and with a passage number of approximately 10. Neuronal differentiation was performed in serum-free Neurobasal medium supplemented with 0.5 mM GlutaMAX (Thermo Fisher Scientific, UK), B-27 supplement (1 ml/50 ml of media), and 1% penicillin/streptomycin (10,000 U/ml). Dibutyryl-cyclic-adenosine-monophosphate (dbcAMP) (Sigma-Aldrich, UK) was used as a differentiator compound and added at a final concentration of 400 µM to the cell cultures.

Differentiated SH-SY5Y cells were treated with 100 µM N-methyl-D-aspartatic acid (NMDA) to activate NMDA glutamate receptors at synaptic sites. Cells were cultured for 24 h and then differentiated for 48 h. NMDA was added and left to incubate at room temperature for either 5 min or 30 min after which the media and agonist were removed, and cells washed. Differentiated activated and non-activated cells were fixed in 4% paraformaldehyde for 15 min and permeabilised in 0.2% Triton-x-100 for 10 min, before blocking using 3% BSA. Cells were incubated with the following diluted primary antibodies: rabbit monoclonal anti-m^6^A (Abcam, ab190886; 1:250); mouse monoclonal anti-ALYREF (Abcam, ab6141; 1:250) and goat polyclonal anti-PSD-95 (Abcam, ab12093; 1:100) at 4 °C. Cells were subsequently incubated with goat Alexa Fluor-conjugated secondary antibodies (Anti-rabbit AF568 [Invitrogen, A10042; 1:500], anti-mouse AF488 [Abcam, ab150105; 1:500] and anti-goat AF647 [Abcam; ab150135]; 1:500). Nuclear staining was performed by adding 1 µg/ml DAPI for 10 min. Coverslips with immunostained cells were mounted using Antifade Fluorescence mounting media.

Cells were visualised on a confocal LSM 710 microscope (Carl Zeiss, Germany). A green channel was excited at 488 nm and emission recorded at 520 nm, and a red channel was excited at 561 nm and emission recorded at 605 nm. A far-red channel was excited at 633 nm and emission recorded at 670 nm. All channels had an emission recording bandwidth of 40 nm. Images were captured at a 16-bit depth using a 63 × Plan-Apo oil objective (NA = 1.4) and with consistent settings: pin hole size = 1 Airy unit; frame size = 1024 × 1024; averaging = 2; and pixel dwell = 3.15 s. Approximately 20 2D single plane images were collected corresponding to 10 images per duplicate coverslips. Negative and positive controls were performed. Quantitative colocalisation analyses were conducted using the Fiji software [85]. ALYREF abundance was quantified in the nucleus and in the cytoplasm before and after synaptic activation. To execute this, the channel corresponding to ALYREF immunofluorescence was background-subtracted and segmented. Regions of interest in the area of the nucleus and cytoplasm were delineated using the Fiji freehand line tools, and the mean pixel gray levels were quantified. Ten image fields were captured, and at least three cells were measured per image. To determine total cytoplasmic colocalisation between m^6^A-modified RNAs and ALYREF immunofluorescence, a nuclear mask was first created using the DAPI ‘nuclear’ channel and smoothened using the Gaussian blur filter before manual thresholding. The whole-cell region of the remaining channels was merged, and the nuclear mask region was subtracted to create a cytoplasmic region of interest. Colocalisation of two channels of interest was subsequently measured within the cytoplasmic region. To measure m^6^A and ALYREF colocalisation specifically at post-synaptic sites, the method followed is described by [14]. Pearson correlation coefficients (PCC) were calculated to measure colocalisation between m^6^A and ALYREF immunoreactivity after two time points, 5 min and after 30 min, and measured within the ‘whole cytoplasm’ and specifically at post-synaptic sites.

### Statistical Analyses

*P*-values of less than 0.05 were accepted as significant in Gene Ontology (GO) analyses performed using DAVID. Confocal immunofluorescence PCC data was subjected to a one-way analysis of variance (ANOVA, two-tailed) with post hoc multiple comparisons, and corrected *p* values < 0.05 were considered significant. Protein network images were produced by using OmicsNet 2.0 [86] using the 2D and 3D auto layout setting. Figures and graphs were generated using GraphPad Prism 8.0.0 for windows (GraphPad Software, California, USA).

## Results

### Reciprocal Modification of m^6^A and m^5^C Effector Protein Transcripts

**Fig. 1:**
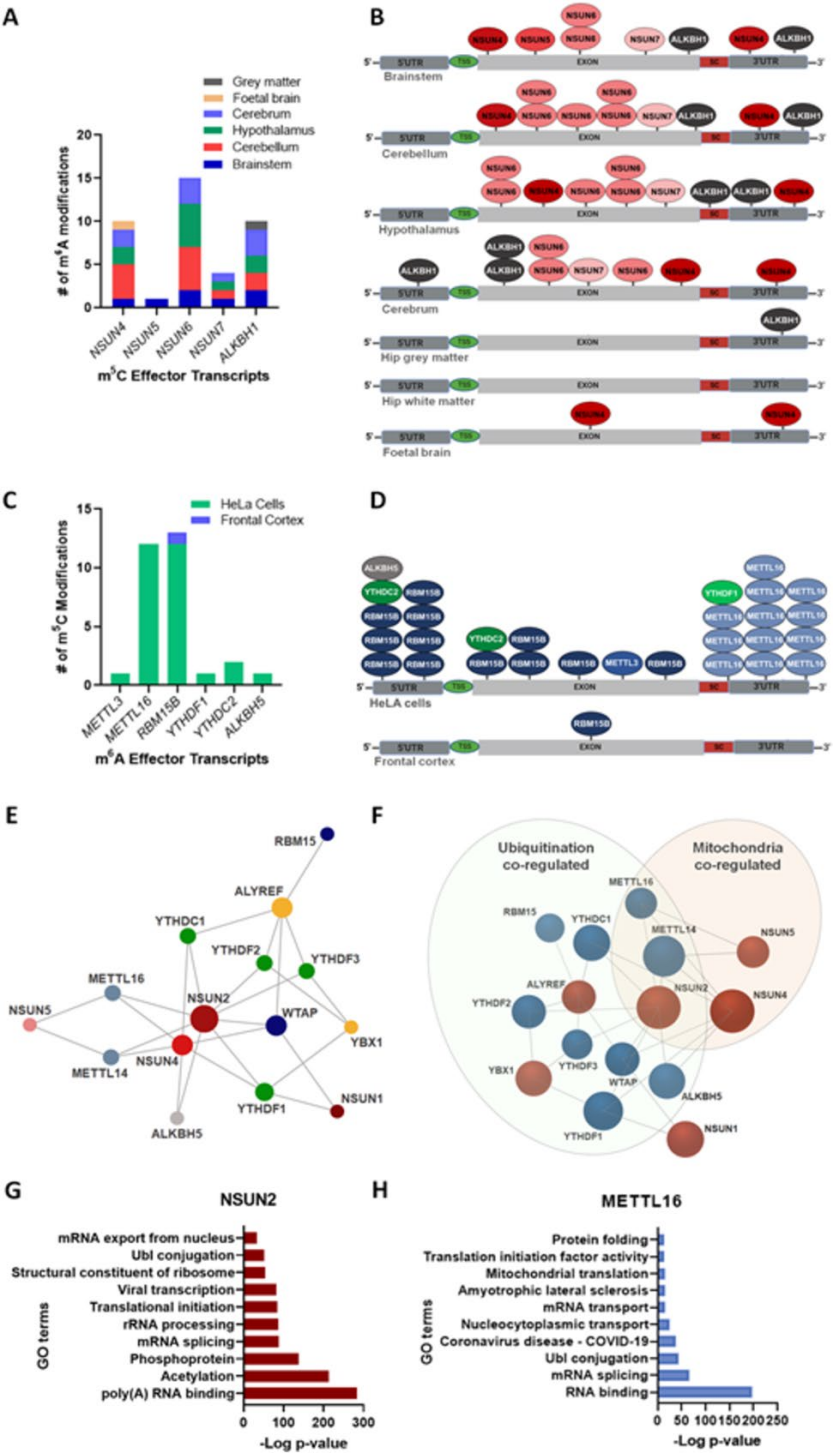
Reciprocal modification of m^6^A and m^5^C effector protein transcripts and co-regulated Gene Ontology terms. **A** m^5^C writer protein transcripts, *NSUN4*, *NSUN5*, *NSUN6* and NSUN*7* and eraser *ALKBH1*, are m^6^A-modified in the brain tissue from the brainstem, cerebellum, hypothalamus, cerebrum, hippocampal grey matter or from foetal tissue. **B** m^6^A modification topology on writer and eraser m^5^C transcripts across the brain regions. m^5^C writers are coloured red and the eraser coloured grey. **C** m^6^A effector protein transcripts *METTL3, METTL14, RBM15B, YTHDF1* and *YTHDC2* and *ALKBH5* are m^5^C-modified in HeLa cells, whereas only *RBM15B* is modified in the frontal cortex*.* **D** m^5^C modification topology is variable along m^6^A-modified effector protein transcripts in HeLa cells. m^6^A writers are coloured blue, readers green and the eraser coloured grey. **E** Visualisation of co-regulated cross-modification system effector protein network. Edges represent co-regulation between node effector proteins with a ProteomeHD percentile score of above 0.8. Multiple edges indicate co-regulation with multiple proteins and larger nodes indicate multiple edges. Red and orange nodes denote m^5^C writer and reader proteins respectively. Blue, green and grey nodes denote m^6^A writer and reader proteins respectively. **F** Schematic of m^5^C and m^6^A co-regulated protein network highlighting m^5^C and m^6^A effector proteins which are associated with mitochondrial function, ubiquitination, or both processes. Red circular node proteins represent m^5^C effector proteins and blue circular modes m^6^A effector proteins. **G** Enriched GO terms for co-regulated proteins with NSUN2 indicating that acetylation, phosphorylation and ubiquitination post-translational modification are co-regulated processes. **H** Enriched GO terms for co-regulated proteins with METTL16 highlighting mitochondria and neurodegenerative disease, amyotrophic lateral sclerosis-associated proteins are co-regulated. Abbreviations: GO, Gene Ontology; SC; stop codon TSS; transcription start site

We previously reported that m^6^A effector proteins were commonly m^6^A multi-modified in the human brain, and hence the m^6^A modification system showed autoregulation [14]. Here, we first examined if m^6^A modifications occur along m^5^C effector protein transcripts and whether this cross-system ‘alloregulation’ is mutual. Using m^6^A-sequencing generated from human brainstem (BS), cerebellum (CER), hypothalamus (HYP) cerebrum (CEREB), parahippocampal grey and white matter as well as foetal brain, we found that m^5^C writer proteins transcripts *NSUN4*, *NSUN5*, *NSUN6*, *NSUN7* and the eraser *ALKBH1* were m^6^A-modified in most brain regions (Fig. 1A). Unlike autoregulation of m^6^A effector proteins which showed the most modified class of m^6^A effector transcripts are the readers, m^5^C reader transcripts were not found to be m^6^A-modified. The observation that m^5^C writers and not readers are m^6^A-modified might suggest some form of hierarchical regulatory relationship between the two pathways. The total number of identified modification sites per transcript across the brainstem, cerebellum, hypothalamus and cerebrum for *NSUN4-7* was 10, 1, 15 and 4, respectively. Of these, *NSUN6*, which methylates tRNA and type II m^5^C mRNAs [47], showed the highest number of different individual m^6^A modification sites across the brain regions. Likewise, the RNA demethylase, *ALKBH1*, also showed 10 single m^6^A modification sites across the brainstem, cerebellum, hypothalamus, cerebrum and hippocampal grey matter.

We next examined the topology of m^6^A modification sites along effector protein transcripts. m^5^C effector transcripts showed variable m^6^A positioning along transcripts. The *NSUN4-7* writers were found to be m^6^A-modified in exonic coding regions within the brainstem, cerebellum, hypothalamus and cerebrum tissue (Fig. 1B). *NSUN4* showed additional m^6^A modification sites located outside exons, namely in the 3’UTR regions, as evident in 4 brain regions (BS, CER, HYP, CEREB) and in foetal brain tissue. Similarly, *ALKBH1* was found to be m^6^A modified within all transcript domains, i.e. exons, 3’UTR, stop codon sites and 5’UTRs in BS, CER, HYP and CEREB brain tissue as well as in the 3’UTR in parahippocampal grey matter. These results suggest there may be tight regulation of where the m^6^A modification occurs along m^5^C writer effector transcripts. Whereas, topological flexibility in m^6^A binding locations evident for *ALKBH1* may reflect the varied functional consequences of ALKBH1 demethylation activity on different RNA species modifications, e.g. tRNAs, mt-tRNAs and mt-m^5^C [49, 87].

To assess if m^6^A effector proteins were subject to reciprocal m^5^C modification, we first examined a m^5^C-seq dataset generated from human HeLa cells. m^5^C modifications were identified only within the m^6^A writer transcripts, *METTL3*, *METTL16* and *RBM15B*; two m^6^A readers, *YTHDF1* and *YTHDC2*; and the eraser, *ALKBH5* (Fig. 1C). The m^6^A writer, *RBM15B*, which has only one exon was highly m^5^C-modified with 12 m^5^C sites predominantly within a short 5’UTR and the single exon but not the larger 3’UTR region (Fig. 1D). Such multi-modification may contribute to the regulation of RBM15B function*,* for example, involvement in RNA positioning during methyl transfer, and nuclear splicing and nuclear export of RNAs [78, 88]. However, of note, RBM15, a paralogue of RBM15B which has similar proposed functional activities [20], was not found to be m^5^C-modified. *METTL16* is also highly modified with 12 m^5^C sites all within the 3’UTR, whereas *METTL3* has one m^5^C modified site within an exon. The two m^6^A readers, *YTHDF1* and *YTHDC2*, showed one or two modification sites per transcript (Fig. 1C, D), and modified sites were within the 5’UTR, exons and 3’UTR regions. The *ALKBH5* eraser transcript also had only one modified region located within the 5’UTR. We next examined m^5^C modification sites in human frontal cortex brain tissue [62]. Only one transcript, *RBM15B*, was found to be m^5^C-modified and with the modified base positioned within the single exon but not at the same bases identified in HeLa cells (Fig. 1D). These observations support that both m^6^A and m^5^C effector proteins are subject to reciprocal RNA modification in brain tissue and human cell cultures but show specific patterns relating to the different effector classes. The variability in the number of modification sites across tissue brain regions may also indicate context-specific tissue and cell type regulation which relates to cellular function.

### Protein Co-regulation of m^6^A and m^5^C Effector Proteins

We hypothesised that changes in m^6^A and m^5^C modifications may be co-regulated during physiological cellular responses to stimuli. To assess whether effector proteins show co-regulation, we used the ProteomeHD software developed to analyse isotope-mass labelling spectrophotometry data generated after biological perturbations to capture relationships between proteins that do not physically interact or colocalise [82]. Table 1 lists m^6^A effector proteins which were found to be co-regulated with m^5^C effector proteins. We observed that m^6^A writers and readers showed similar and consistent co-regulation patterns with specific m^5^C effector proteins (Fig. 1E). For example, the m^6^A writer protein, RBM15B, and m^6^A writer methylase complex adapter, WTAP, are both co-regulated with the m^5^C reader *ALY/REF* export factor (ALYREF). As RBM15B, a second member of the RNA-binding motif protein 15 family of proteins, is reported to interact with METTL3 in a WTAP-dependent fashion [88], as well as being involved in nuclear export of mRNAs to the cytoplasm within the TREX complex in which ALYREF is a component [78], their co-regulation is perhaps unsurprising. However, of interest, METTL3 was not identified as being co-regulated with RBM15, WTAP and ALYREF or indeed with METTL14. This observation provides some support for METTL3 having an alternative ‘moonlighting’ role within mammalian cells. In addition, the m^6^A writers METTL14 and METTL16 were highly co-regulated with the m^5^C writer proteins, NSUN4 and NSUN5, as well as METTL16 with NSUN2. The m^6^A readers, YTHDF1-3, and YTHDC1, were commonly found to be co-regulated with three m^5^C effector proteins, the writer NSUN2, and the m^5^C readers YBX1 and ALYREF. The co-regulation grouping for the m^6^A readers is intriguing and suggests that cross talk between these effector systems is part of an established cellular response process.

Four m^5^C effector proteins, NSUN1, NSUN2, NSUN4 and ALYREF, were observed to have m^6^A effectors proteins as being co-regulated (Table 1, Fig. 1E). The m^5^C writer NSUN2 which methylates various RNA species, e.g. tRNAs and mRNAs, was indicated to have the highest number of m^6^A co-regulation partners and which included writer, reader and the ALKBH5 eraser proteins. ALYREF also showed broad co-regulation with m^6^A writers and readers, and commonly co-regulated to both NSUN2 and ALYREF were the m^6^A effector proteins WTAP, YTHDC1, YTHDF2 and YTHDF3. Finally, the rRNA mitochondrial writer NSUN4 showed co-regulation with the eraser ALKBH5. As ALKBH5 has not as yet been associated with mitochondrial methylation or rRNA modification, this suggests that NSUN4 or ALKBH5 may have more diverse functional activity than is currently known. Together, these observations suggest that: the two RNA modification systems are co-ordinately co-regulated after biological perturbation; the m^6^A readers are associated with a specific subset of m^5^C effector proteins; ALYREF has the most and diverse co-regulation effector partners; and that ALKBH5 and NSUN4 have molecular activities which are as yet unidentified.

### Gene Ontology Analysis of the Protein Co-regulation Partners of m^6^A and m^5^C Effector Proteins

By performing Gene Ontology analysis, we next examined the biological functions of co-regulated m^6^A and m^5^C proteins. As might be expected, enriched GO terms common to all co-regulated m^6^A and m^5^C effector proteins were RNA binding or poly(A) RNA binding, protein binding and splicing whether specific to mRNA or other RNA species. However, some common cellular processes found significantly and highly enriched appeared specific to subsets of effector proteins which belonged to both m^6^A and m^5^C modification systems. For instance, the terms phosphorylation, SUMOylation and ubiquitination all relate to post-translational modification of proteins and showed high levels of enrichment and moderate size of effects, e.g. phosphorylation (*p* < 3.7 × 10^−152^ to *p* < 5.2 × 10^−17^, fold enrichment 1.4–2.0); SUMOylation (*p* < 7.2 × 10^−24^ to *p* < 1.5 × 10^−4^, fold enrichment 2.8–6.8); ubiquitination (*p* < 1.1 × 10^−62^ to *p* < 6.5 × 10^−3^, fold enrichment 2.2–5.7). Nevertheless, enrichment for the terms phosphorylation or phosphoprotein was only evident in a subset of m^6^A effector proteins which included the m^6^A effector proteins WTAP, RBM15, METTL16, YTHDC1, YTHDF1, YTHDF3, ALKBH5 and m^5^C effector proteins NSUN1, NSUN2, NSUN5 and YBX1 (Table S2). Of note, YTHDF2 is not included along with YTHDF1 and YTHDF3. The highest percentage overlap of phosphorylation-associated co-regulated proteins across the two methylation systems was for ALKBH5 with NSUN2 (79.5%; ALKBH5 233/293, NSUN2 total 767) and NSUN1 and WTAP (73.9%; NSUN1 435/589, WTAP total 784).

In a similar fashion, enrichment for SUMOylation processes was evident for only METTL14, WTAP, RBM15, YTHDC1, YTHDF1, YTHDF2, ALKBH5, NSUN1-5 and ALYREF (Table S3). As YTHDF3 was not enriched for SUMOylation processes, again a difference between the YTHDF1-3 readers was apparent. In general, although much lower in the total number of SUMOylation-associated proteins than for the term phosphorylation, the highest overlap of common co-regulated proteins was observed between NSUN1 and WTAP (NSUN1/WTAP 93.8%, NSUN1 15/16, WTAP total 31); NSUN1 and RBM15 (NSUN1/RBM15 81.3% NSUN1 13/16, RBM15 total 44); and NSUN1 and YTHDC1 (NSUN1/YTHDC1 87.5%, NSUN1 14/16, YTHDC1 total 44). Shared co-regulated SUMOylation-related proteins was also evident between ALKBH5 with NSUN2 (86.7%; ALKBH5 13/15, NSUN2 total 34) and NSUN2 and YTHDC1 (NSUN2/YTHDC1 88.2%; NSUN2 30/34, YTHDC1 total 44).

Likewise, co-regulated proteins associated with the term ubiquitination were found enriched for the effector proteins METTL16, WTAP, RBM15, YTHDC1, YTHDF1, YTHDF2 and YBX1, and to a lesser extent, METTL14, YTHDF3, ALKBH5, NSUN2 and ALYREF. The number of ubiquitination-associated co-regulated proteins for the m^5^C system effector proteins was much lower than the majority of ubiquitination-enriched m^6^A effector proteins suggesting that the m^5^C modification system machinery may be less involved in ubiquitination pathways. However, a relatively high number of shared co-regulated proteins across the methylation systems were observed between YBX1 (315 total ubiquitination-associated proteins) and the m^6^A effector proteins, METTL16 (150), WTAP (143), RBM15 (115), YTHDC1 (113), YTHDF2 (161), as well as between NSUN2 (84 total ubiquitination-associated proteins) and METTL16 (56%, *N* = 47), YTHDF3 (40), and ALYREF (38) (Table S4 and Fig. 1F, G). Of interest, the majority of proteasome subunit proteins belonging to the 19S proteasome activator regulatory particle (e.g. 19S PSMC and PSMD subunit proteins) and proteasome S20 core particle (e.g. PSMA and PSMB subunit proteins) were co-regulated with METTL14, METTL16, NSUN2, ALYREF and YBX1 proteins (Table S5). However, although PSMA and PSMB particle subunits were co-regulated with WTAP, RBM15, YTHDC1, YTHDF1, YTHDF2 and YTHDF3, the 19S proteasome regulatory particle lid PSMD subunit proteins were not. This observation might suggest that distinct proteasome subunit components and hence protein degradation processes are differentially co-regulated with RNA methylation effector proteins.

Terms relating to mitochondria-specific processes also showed distinct enrichment patterns across the effector protein systems. For example, mitochondrial GO terms were found to be significantly enriched for co-regulated proteins associated with METTL14, METTL16, NSUN2, NSUN4 and NSUN5 (Fig. 1F and H and Table S6). Of these effector proteins, only the m^5^C system writers, NSUN2, NSUN3 and NSUN4, are recognised to be mitochondria methyltransferases with NSUN2 involved in the generation of m^5^C at positions 48, 49 and 50 of mammalian mt-tRNAs [89], while NSUN3 methylates position 34 of mt-tRNAs [49, 51], and NSUN4 is reported to be involved in both methylation of mitochondrial 12S rRNA and mitoribosomal assembly [50, 60, 90]. However, IME4, an m^6^A writer in yeast, when deleted in Saccharomyces cerevisiae, causes mitochondrial dysfunction indicating that proteins within the m^6^A modification system may also have a key role in mitochondrial RNA processes [91]. Indeed, across the modification systems, METTL14 and METTL16 have the highest overlap in co-regulated mitochondrial-associated proteins with NSUN4 (METTL14/NSUN4 81.3%, METTL14 169/208, NSUN4 total 372; METTL16/NSUN4 77.3%, METTL16 136/176, NSUN4 total 372) and showed enrichment for the mitochondrial terms specific for mitochondrial compartments, e.g. mitochondrial matrix or inner membrane, as well as mitochondrial translation and mitochondrial small ribosomal subunit function.

NSUN2 is known to cause forms of autosomal recessive intellectual disability (AR ID) [92–95] as are methyltransferases from other modification systems, e.g. *FTSJ1*, a human tRNA 2′-O-methyltransferase [96], and *METTL5*, an N^6^ adenine DNA and rRNA writer [30, 97]. We observed that intellectual disability (ID), also known as mental retardation, was an enriched term for co-regulated proteins for several of the m^6^A and m^5^C methylation effector proteins, namely, METTL16, WTAP, RBM15, YTHDC1, YTHDF1, YTHDF2, ALKBH5, NSUN1, NSUN2, NSUN4, NSUN5 and YBX1. METTL16 had the highest overall number of intellectual disability co-regulated proteins with 55 and was the only effector protein which had NSUN2 listed as an ID co-regulated protein. Nevertheless, the highest overlap both in terms of percentage and actual number of shared co-regulated proteins between methylation systems was for WTAP, RBM15, YTHDC1 and YTHDF1 with NSUN1 and NSUN2 (Table S7). Common to these sets of m^6^A and m^5^C effector proteins were the proteins MED23 and MED25 (Tables S8, S9) which cause AR ID, syndromic ID and eye–intellectual disability syndrome [98–100] and which are components of the mediator complex which repress transcription by RNA polymerase II. However, NSUN1 and WTAP, RBM15, YTHDC1 and YTHDF1 are also commonly co-regulated with MED12, THOC2 and or THOC6 (Table S8). Again, MED12 is involved in transcription activation and mutations within the gene cause a variety of X-linked intellectual disorders with dysmorphic features [101], whereas THOC2 and THOC6 encode subunits of the TREX mRNA-export complex which couples mRNA transcription, processing and nuclear export and are reported to cause X-Linked ID and rare AR syndromic ID [102, 103]. These findings suggest that NSUN1 and NSUN2 and the specific writer and reader proteins detailed above may share involvement in co-regulated ID-associated mechanisms, but NSUN1 and the m^6^A effector proteins may also co-function in NSUN2-independent ID pathways.

### In Vivo Colocalisation of m^6^A modified RNAs with the m^5^C Reader Protein ALYREF

**Fig. 2:**
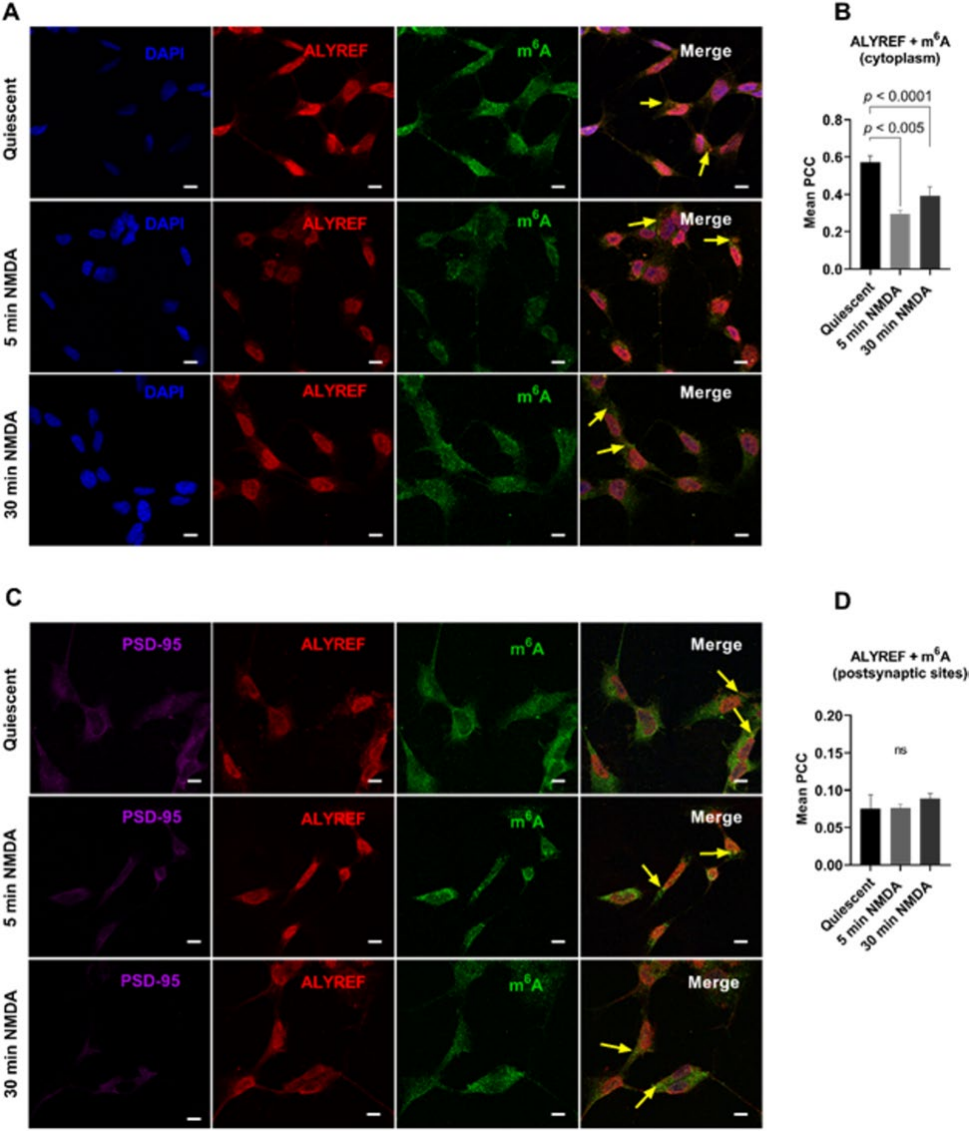
Colocalisation between the m^5^C reader ALYREF and m^6^A-methylated RNAs within the cytoplasm and post-synaptic sites in differentiated neuronal cells before and after NMDA synaptic activation. **A** Single plane images of dSHSY5Y cells showing colocalisation of ALYREF and m^6^A-RNAs in the cytoplasm when cells are quiescent, and after NMDA synaptic activation at time 5 min and time 30 min. **B** Mean Pearson’s correlation coefficients for ALYREF and m^6^A-RNAs in the cytoplasm when cells are quiescent, and after NMDA synaptic activation at times 5 min and 30 minu. A significant increase in ALYREF and m^6^A-RNA colocalisation after NMDA activation is evident after 5 min (*p* < 0.005) and 30 min (*p* < 0.0001). **C** Single plane images of dSHSY5Y cells showing colocalisation of ALYREF and m^6^A-RNAs at post-synaptic sites when cells are quiescent, and after NMDA synaptic activation at time 5 min and time 30 min. **D** Mean Pearson’s correlation coefficients for ALYREF and m^6^A-RNAs at post-synaptic regions when cells are quiescent, and after NMDA synaptic activation at times 5 min and 30 min. Yellow arrows point to regions of colocalisation within the cytoplasm. Cells were prepared in duplicates and ten images per cell were collected giving a total of 20 images per labelled combinations. Scale bar = 50 µm

To explore how, and if, m^6^A and m^5^C methylation processes could be co-regulated in a spatial and temporal manner and after biological stimulation in vitro, we examined the relationships between m^6^A-modified RNA and the m^5^C reader protein, ALYREF, in differentiated neuronal SHSY5Y (dSHSY5Y) cells. We quantified colocalisation between m^6^A-RNAs and ALYREF within the cytoplasm when cells were quiescent and after treatment with NMDA to activate NMDA receptors at synapses. As we have previously reported changes in m^6^A-RNA abundance colocalising with YTHDF1, YTHDF3 and ALKBH5 after synaptic activation at synaptic sites, we also examined m^6^A-RNAs and ALYREF colocalisation in post-synaptic regions. dSHSY5Y cells were assessed at three time points: no activation (quiescent); 5 min after activation (reflecting early synaptic plasticity); 30 min after activation (later-stage early plasticity). m^6^A-modified RNAs and ALYREF were both found abundant in the cytoplasm with ALYREF showing more expression in, and surrounding, the nucleus (Fig. 2A). In differentiated quiescent dsSHSY5Y cells, ALYREF and m^6^A-modified RNAs were found to be highly colocalised in the cytoplasm, PCC 0.57 ± 0.04. However, colocalisation within the cytoplasm significantly decreased after synaptic activation both at the very early stage of plasticity (5 min, PCC 0.29 ± 0.02, *p* < 0.0001) and later-stage plasticity (30 min, PCC 0.39 ± 0.05, (*p* < 0.005) (Fig. 2B).

ALYREF is known to be part of the TREX complex that shuttles mRNA out of the nucleus [104]. To assess if this decrease in colocalisation between ALYREF and m^6^A-modified RNAs may relate to changes in ALYREF localisation within cell sub-compartments after synaptic activation, we quantified ALYREF abundance in the nucleus and cytoplasm in quiescent and NMDA-activated cells. ALYREF abundance in the cytoplasm was found to be significantly increased (*p* < 0.0001) after 5-min NMDA activation (mean grey values 6214 ± 336) and (*p* < 0.0001) after 30-min NMDA activation (mean grey values 5806 ± 313), compared to levels in non-activated dsSH-SY5Y cells (mean grey values 3913 ± 233) (Fig. S1). There were no significant differences in ALYREF cytoplasmic abundance between the 5- and 30-min activation states (*p* > 0.05). These observations are consistent with ALYREF translocating to the cytoplasm after synaptic activation. The findings also indicate that although ALYREF abundance increases in the cytoplasm with NMDA activation, colocalisation with modified RNAs decreases, suggesting a negative relationship between ALYREF and m^6^A-modified RNAs within the cytoplasm after synaptic activation. We also observed that ALYREF is present at post-synaptic sites. However, in non-activated dsSHSY5Y cells, ALYREF and m^6^A-methylated RNA showed low colocalisation, PCC 0.076 ± 0.02, and after synaptic activation, no difference in colocalisation was observed (5 min, *p* = 0.99, PCC 0.076 ± 0.005; 30 min,* p* = 0.70, PCC 0.089 ± 0.007) (Fig. 2D). This finding suggests that there is a lack of a functional relationship between the m^5^C reader ALYREF and m^6^A methylated RNAs after synaptic activation at post-synaptic sites.

Finally, ALYREF has been recently suggested to interact with the m^6^A demethyltransferase, ALKBH5, in primary human hepatocytes [105]. To substantiate our findings that ALYREF has a putative functional relationship with a broader range of m^6^A modification proteins in both the nucleus and the cytoplasm, we used the BioGRID database to identify proteins interacting with ALYREF. We noted that in a total of 15 studies which used co-fractionation, affinity capture-MS, affinity capture-western or proximity label-MS [67–81], and which identified ALYREF binding partners, ALYREF was reported to interact with three m^6^A nuclear writer proteins (METTL14, RBM15, RBM15b), and fifteen nuclear and/or cytoplasmic proteins identified as preferentially interacting with modified (CPSF6, IGF2BP3, SF3B4, XRN1) or unmodified (BRD7, CHD3, HDLBP, INO80b, PCF11, RBM42, REST, SRSF1, TRIM25, UBE2I, ZC3HAV1) m^6^A-RNA binding sequences. These observations add further evidence of a direct protein–protein physical interaction between the RNA modification systems although whether the outcome of such interaction is synergistic or antagonistic remains to be determined on an individual basis.

## Discussion

The m^6^A and m^5^C modification systems have conventionally been thought to be independent from one another although the same single RNA transcript might be modified with both forms of modification. Recent studies have, however, provided evidence of m^6^A and m^5^C methylation systems acting synergistically to enhance methylation along specific single transcripts [59, 60]. Similarly, two studies, which both focused on changes in gene expression as a means of predicting cancer prognosis [106, 107], found a clustering of mRNA expression changes of effector transcripts involved in m^6^A, m^5^C, m^1^A and m^7^G modification as well as proteins involved in other post-transcriptional processing, e.g. A to I editing RNA proteins, with different colorectal or soft-tissue sarcoma tumour types. As these clusters of expression changes associated with prognosis involved effector transcripts across the modification types, the authors proposed that there exists cross talk between RNA modification regulators. Furthermore, it has been recently reported that the interaction of two effector proteins, YTHDF2 and HSRP12, for m^6^A and m^1^A methylation systems, respectively, enhance mRNA degradation, and that transcripts which are both m^6^A and m^1^A modified are downregulated in an HRSP12-dependent manner compared with mRNAs modified with m^6^A only [108]. In addition, a study, which utilised machine learning techniques of Oxford Nanopore RNA direct sequencing to predict m^6^A and pseudouridine modification sites, revealed an opposing transcriptomic co-occurrence of m^6^A and pseudouridine modification, and synergistic, hierarchical effects of m^6^A and pseudouridine on the polysome [109]. Such recent studies provide significant support for the potential for widespread interaction between RNA modification systems.

Here, we provide evidence at a global scale that the m^6^A and m^5^C RNA methylation systems regulate each other’s activity through cross modification of effector protein transcripts. We revealed that the functional consequence of cross-system control on co-regulated proteins shows variation across processes occurring in sub-compartments and acting upon different species of RNA. Our findings also corroborate the results of functional studies that have previously interrogated the involvement of individual effector proteins in cellular mechanisms. For example, we provide substantiating evidence that the m^6^A YTHDF1-3 reader proteins may have distinct roles in RNA processing and cellular pathways and that such processes are contingent on protein subcellular expression. We also provide clear evidence for new putative molecular roles for well-studied RNA-binding proteins and interaction between methylation systems involving specific subsets of proteins, for example the m^6^A writer METTL16 and a potential role in mitochondrial processes and the YBX1 reader and association with ubiquitination and proteasome degradation proteins. Furthermore, we revealed a co-regulatory relationship between m^6^A ALKBH5 mRNA eraser and rRNA mitochondrial m^5^C writer, NSUN4, which share common enriched processes such as rRNA processing, RNA splicing and acetylation. Such novel observations highlight that we are still at a discovery stage of understanding RNA effector protein function and consequences upon biological activity.

Co-regulation between methylation systems after biological perturbation could manifest as either a positive or a negative relationship. In our in vitro findings, we demonstrated a significant decrease in ALYREF colocalisation with abundance of m^6^A-modified RNAs in the cytoplasm after synaptic activation compared to the cell quiescent state even though ALYREF abundance significantly increased in cytoplasmic regions after synaptic stimulation. This observation is consistent with a negative relationship between ALYREF and modified RNAs in the cytoplasmic region following synaptic activation. As ALYREF is part of the TREX complex which is reported to transport m^6^A-modified RNA [104], the relationship of this m^5^C reader may not involve direct binding to m^6^A-RNAs. Indeed, the resolution of confocal immunofluorescence colocalisation is spatially low and should not be taken as direct evidence of physical interaction. However, a clear relationship, whether a positive or negative correlation, between post-translational modification (PTM) mechanisms and specific subsets of m^6^A and m^5^C effector proteins was evident and provides complementary evidence implicating that proteins which are involved in protein phosphorylation are highly m^6^A modified in white and grey matter brain tissue [14]. Furthermore, the newly apparent relationship between co-regulated proteins involved in ubiquitination and the observed differences between proteasome subunit components suggest that degradation processes that are important, for example, in eliminating toxic misfolded proteins [110] may differentially involve subsets of cross-system methylation effector proteins. Future mass spectrometry studies of knockdown or knockout m^6^A and m^5^C effector proteins examining changes at the cellular, sub-compartment and nano-domain level will be important for elucidating the relationship, and direction of relationship, of RNA modification mechanisms in proteasome processes.

Our findings also highlight known and novel relationships between effector proteins and disease. To date, only NSUN2, NSUN3 and NSUN4 are known to be involved in methylation of rRNA and tRNAs in mitochondria, and mutations in mt-tRNA m^5^C RNA writer, *NSUN3*, are already recognised to cause mitochondrial disease [89, 111]. Here, we provide new evidence that in addition to m^5^C writer proteins, the m^6^A writers METTL14 and METTL16 may act as mitochondria RNA methyltransferases. The high co-regulation of proteins with NSUN4 would suggest a putative role in the transfer of methyl groups onto rRNAs. However, for both METTL14 and METTL16, mitochondrial terms relating to mitochondrial compartments, e.g. mitochondrial matrix or inner membrane, as well as mitochondrial translation and mitochondrial small ribosomal subunit function, were enriched suggesting a possible broader function within mitochondria. Whether they cause or contribute to the development of mitochondrial disease or complex diseases where mitochondria dysfunction is part of the disease process such as in neurodegenerative disorders [112] is yet to be examined. Similarly, mutations within the mRNA and tRNA m^5^C writer *NSUN2* cause neurodevelopmental disorders such as autosomal recessive intellectual disability (AR ID), and loss-of-function mutations within methyltransferases involved in tRNA and rRNA modification at alternative bases cause various forms of intellectual disability [30, 96, 113]. Our findings suggest that several of the writer and reader m^6^A and m^5^C methylation effector proteins including NSUN2 are part of a co-regulated protein cellular response which shows changes with known ID disease-causing proteins such as MED and THOC proteins. However, the underlying cellular mechanisms reported in previous studies which may be contributing to neuronal dysfunction in ID appear diverse and could involve transcription repression or activation, mRNA processing and nuclear export [114–116]. How changes in modification of rRNA, tRNAs and mRNAs regulated by both m^5^C and m^6^A systems contribute to such disrupted cellular processes during distinct developmental stages have still to be determined but may lead to new therapeutic molecular targets for ID neurodevelopmental disorders.

This study has provided a valuable new understanding of processes governing RNA metabolism and coordinated cellular responses. Nonetheless, many questions remain in this fast-emerging, stimulating field. We still have limited knowledge of what the consequences are for m^5^C and m^6^A modifications existing at close proximity along transcripts or different RNA molecules. Do effector proteins bind to their respective modification base site and thereby block or repel other modification RNA-binding proteins or form complexes which interact with the alternative modification system effector proteins? Proteins have already been identified as preferentially interacting with unmodified m^6^A-RNA binding sequences, i.e. they are repelled by ‘m^6^A’ RNA modifications [66, 117], and very recently, it has been shown that m^6^A specificity is globally regulated by ‘suppressors’ that prevent m^6^A deposition in unmethylated mRNA transcriptome regions [118]. Such suppression of sites appears to involve suppression of m^6^A deposition rather than active demethylation and, as of yet, is associated with changes in splicing. It remains unexplored whether m^6^A suppressed regions have a consequence on translation or degradation processes and hence have a broader impact on cellular behaviour and whether such sites have a high abundance of modified m^5^C bases or are commonly bound by m^5^C machinery. Indeed, we are still at a stage of characterising and defining effector modification proteins, and we continue to evolve new terminology to reflect advances in understanding of RNA methylation mechanisms. The continuing development of nanopore DRS technology and software, with the potential ability to call different forms of RNA modifications at a single base resolution and simultaneously quantify transcript expression, will be important for determining functional consequences specific to cellular environments and changes in m^5^C and m^6^A modification profiles in disease.

## Supplementary Information

Below is the link to the electronic supplementary material.

Supplementary file1 (PDF 328 KB)

Supplementary file2 (XLSX 66.1 KB)

## Data Availability

The analysed datasets used during the current study are available from the corresponding author on reasonable request.
