# Supplementary material for: Global Co-regulatory Cross Talk Between m6A and m5C RNA Methylation Systems Coordinate Cellular Responses and Brain Disease Pathways
Source: Mol Neurobiol. 2024 Nov 5;62(4):5006–21. doi: 10.1007/s12035-024-04555-0 (PMC11880056; doi:10.1007/s12035-024-04555-0)
Supplement: Supplementary file 1 — Supplementary file1 (PDF 328 KB) [file 12035_2024_4555_MOESM1_ESM.pdf]

Supplementary Information: Global co-regulatory cross talk between $m^6A$ and $m^5C$ methylation RNA systems coordinate cellular responses and brain disease pathways.

Oliver Chukwuma Orji[1], Joseph Stones[1], Seema Bagia[2], Robert Markus[2], Merve Demirbugen öz[3],

Helen Miranda Knight[1*]

[1]Division of Cells, Organisms and Molecular Genetics, School of Life Sciences, University of Nottingham, Nottingham, NG7 2UH, UK.

[2]School of Life Sciences Imaging facility, University of Nottingham, Nottingham, NG7 2UH, UK.

[3]Department of Pharmaceutical Toxicology, Faculty of Pharmacy, Ankara University, Turkey.

Supplementary Information: Global co-regulatory cross talk between $m^6A$ and $m^5C$ methylation RNA systems coordinate cellular responses and brain disease pathways.

| **$m^5C$ effectors transcripts** | | **Transcript co-ordinates** | **$m^6A$ modified** |
|---|---|---|---|
| $m^5C$ writers | | | |
| | *NSUN1* | *Chr12:6,666,036-6,677,498* | Not modified |
| | *NSUN2* | *Chr5:6,599,352-6,633,473* | Not modified |
| | *NSUN3* | *Chr5:6,599,352-6,633,473* | Not modified |
| | *NSUN4* | *Chr1:46,805,849-46,830,824* | Multi-modified |
| | *NSUN5* | *Chr1:46,805,849-46,830,824* | Modified |
| | *NSUN6* | *Chr10:18,834,264-18,940,550* | Multi-modified |
| | *NSUN7* | *Chr4:40,751,914-40,812,002* | Multi-modified |
| | *DNMT2* | *Chr10:17,184,982-17,244,070* | Not modified |
| $m^5C$ readers | | | |
| | *YBX1* | *Chr1:43,148,066-43,168,020* | Not modified |
| | *ALYREF* | *Chr17:79,845,711-79,849,462* | Not modified |
| $m^5C$ eraser | | | |
| | ALKBH1 | *Chr14:78,138,749-78,174,356* | Multi-modified |
| **$m^6A$ effectors transcripts** | | | **$m^5C$ modified** |
| $m^6A$ writers | | | |
| | *METTL3* | *Chr14:21,966,282-21,979,457* | Modified |
| | *METTL14* | *Chr4:119,606,574-119,632,077* | Not modified |
| | *METTL16* | *Chr17:2,319,348-2,415,200* | Multi-modified |
| | *WTAP* | *Chr6:160,148,030-160,177,352* | Not modified |
| | *RBM15* | *Chr1:110,881,945-110,889,303* | Not modified |
| | *RBM15B* | *Chr3:51,428,699-51,435,336* | Multi-modified |
| $m^6A$ readers | | | |
| | *YTHDF1* | *Chr20:61,826,782-61,847,538* | Modified |
| | *YTHDF2* | *Chr1:29,063,133-29,096,287* | Not modified |
| | *YTHDF3* | *Chr8:64,081,112-64,125,346* | Not modified |
| | *YTHDC1* | *Chr4:69,176,105-69,215,824* | Not modified |
| | *YTHDC2* | *Chr5:112,849,391-112,930,984* | Multi-modified |
| $m^6A$ eraser | | | |
| | *ALKBH5* | *Chr17:18,086,867-18,113,267* | Modified |

Supplementary Information: Global co-regulatory cross talk between $m^6A$ and $m^5C$ methylation RNA systems coordinate cellular responses and brain disease pathways.

**Supplementary Table 1.** $m^5C$ and $m^6A$ effector protein transcripts examined for modification by the alternative methylation system. Effector protein name, main transcript co-ordinates and categorisation of whether multi modified, modified or not modified in the brain and HeLa cell RNA-seq datasets. Co-ordinates relate to hg19 build.

Supplementary Information: Global co-regulatory cross talk between $m^6A$ and $m^5C$ methylation RNA systems coordinate cellular responses and brain disease pathways.

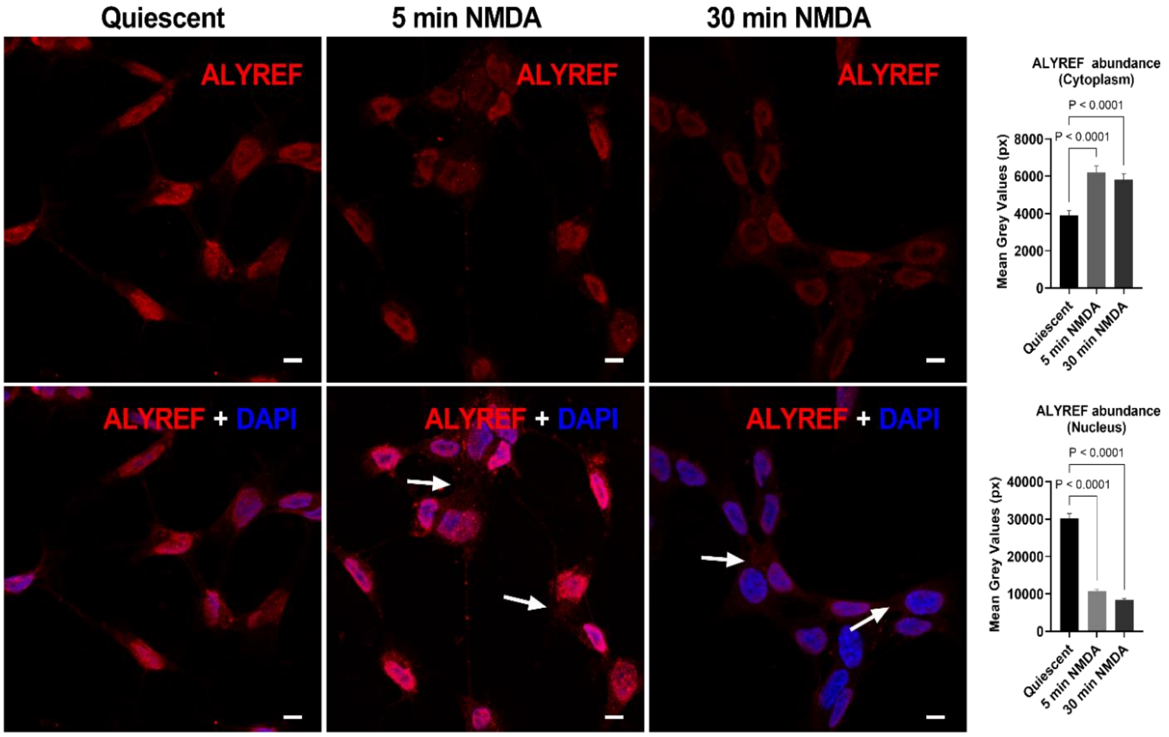


**Supplementary Figure 1. The m5C reader ALYREF abundance in the nucleus and cytoplasm after NMDA synaptic activation in differentiated neuronal cells.** Single plane images of dSHSY5Y cells showing ALYREF in the nucleus and cytoplasmic regions when cells are quiescent, and after NMDA synaptic activation at time 5 minutes and time 30 minutes. White arrows point to regions with high ALYREF cytoplasmic abundance after synaptic activation. ALYREF abundance significantly increases in the cytoplasm after NMDA synaptic activation at times 5 minutes and 30 minutes ($p < 0.0001$). Top row, ALYREF no DAPI; bottom row, ALYREF with DAPI, Scale bar = 50 µm.
